# A novel construct of anhedonia revealed in a Chinese sample via the Revised Physical and Social Anhedonia Scales

**DOI:** 10.1186/s12888-020-02900-w

**Published:** 2020-11-09

**Authors:** Qiongqiong Wu, Jiayue He, Shulin Fang, Panwen Zhang, Xingwei Luo, Jianghua Zhang, Yan Xiong, Fusheng Luo, Xiaosheng Wang, Shuqiao Yao, Xiang Wang

**Affiliations:** 1 Medical Psychological Center, the Second Xiangya Hospital, Central South University, Changsha, 410011 China; 2 Student Affairs Department, Central South University, Changsha, 410083 China; 3 Student Affairs Department, Central South University of Forestry and Technology, Changsha, 410004 China; 4 Department of Human Anatomy and Neurobiology, Xiangya School of Medicine, Central South University, Changsha, 410013 China; 5 Medical Psychological Institute of Central South University, Changsha, 410011 China; 6 National Clinical Research Center for Mental Disorders, Changsha, 410011 China

**Keywords:** Anhedonia, Revised Physical Anhedonia Scale (RPAS), Revised Social Anhedonia Scale (RSAS), Factor structure, Second-order hierarchical model, Chinese version

## Abstract

**Background:**

Anhedonia is a core clinical symptom of mental disorders. The Revised Physical Anhedonia Scale (RPAS) and the Revised Social Anhedonia Scale (RSAS) have been applied in clinical and non-clinical samples since 1980s. However, the construct of a unified RPAS&RSAS for comprehensive measurement of anhedonia has never been explored. Therefore, the purpose of our study was to examine the factor structure of the unified RPAS&RSAS among undergraduates and clinical patients.

**Methods:**

A total of 3435 undergraduates from two universities and 294 clinical patients with mental disorders had completed the Chinese version of the RPAS and the RSAS. Exploratory factor analysis (EFA) and confirmatory factor analysis (CFA) were each conducted to reveal the constructs of the RPAS and the RSAS. CFA was used to evaluate first- and second-order models for the unified RPAS&RSAS in undergraduates and clinical patients. The internal consistency and test-retest reliability of the RPAS and the RSAS were also evaluated.

**Results:**

EFA and CFA indicated 2-factor structures for RPAS and RSAS, with the factors being defined as anticipatory anhedonia and consummatory anhedonia. The second-order model of the unified RPAS&RSAS in the undergraduates and clinical patients both had satisfactory fit index values (Undergraduate sample: CFI = 0.901, TLI = 0.899, RMSEA = 0.055, SRMR = 0.086; Clinical sample: CFI = 0.922, TLI = 0.911, RMSEA = 0.052, SRMR = 0.078). The psychometric robustness of the RPAS&RSAS were confirmed by high internal consistency and test-retest reliability values.

**Conclusions:**

The unified RPAS&RSAS with a second-order structure was confirmed in both undergraduates and clinical samples in Chinese. The construct of anhedonia was refreshed as covering physical and social domains, and each of them includes both anticipatory and consummatory components.

## Background

Anhedonia, defined as a diminished or absent ability to experience pleasure in normally pleasurable daily activities [1], has been associated with a neurotransmitter imbalance in reward processing circuitry involving multiple neuroanatomical areas [2–4] and dopamine dysregulation [3, 5, 6]. Conceptually, anhedonia has been suggested to be composed of two major components, namely anticipatory anhedonia and consummatory anhedonia [7]. Anticipatory pleasure is generated by reward motivation and behaviors aimed at future rewards (i.e.,“wanting”) while consummatory pleasure refers to pleasure in the present moment and reward attainment (i.e., “liking”) [7]. Furthermore, results obtained from the Research Domain Criteria project, whose aim is to integrate information from genomics and circuits to behavior and self-reports in psychiatric disorders [8, 9], suggested that anhedonia may involve two domains, a Negative Valence Systems domain and a Social Processes domain (see www.nimh.nih.gov/research-priorities/rdoc/index.shtml).

Anhedonia is experienced as a symptom of a number of mental disorders [10, 11], including major depressive disorder [12–16], post-traumatic stress disorder [11], obsessive-compulsive disorder [17], schizoid and schizotypal personality disorder [18, 19], and schizophrenia (as a negative symptom) [1, 20–23]. Therefore, there is a need for assessment and differential diagnosis with instruments that are not specific to a particular diagnosis.

The Chapman psychosis-proneness scales are series of scales that were designed to assess schizotypy tendencies in psychiatrically normal people [19, 24–27]. Four of the scales in this series, namely the Perceptual Aberration Scale, Magical Ideation Scale, Physical Anhedonia Scale, and Social Anhedonia Scale, form a schizotypy assessment battery known as the Wisconsin schizotypy scales (WSS). In recent years, the revised versions of the two anhedonia scales within the WSS, known as the Revised Physical Anhedonia Scale (RPAS) and the Revised Social Anhedo’nia Scale (RSAS), have been used alone across different cultures [28–31] and in various kinds of sample populations [19, 32, 33]. The RPAS assesses the lack of pleasure experienced in physical sensations, whereas the RSAS assesses the lack of pleasure in the social realm [34].

The RPAS and the RSAS are broadly appreciated for the inclusive information they cover, and their simple format, which requires participants to answer true/false questions. Testing of the psychometric robustness of the RSAS and RPAS has shown they both have good reliability (RPAS α = 0.71–0.93, RSAS α =0.82–0.95; r_test-retest_ = 0.74–0.87) and good validity in the USA [1, 35], France [36], Germany [37, 38], Turkey [31], Spain [29] and China [30] (see Table 1). Although earlier the RSAS/RPAS studies involved primarily clinical samples, recent studies have focused on non-clinical populations [39]. For example, Chan et al. showed that the RPAS and RSAS, which are self-report scales used to assess anhedonia severity across multiple neuropsychiatric stages [40], could be used to examine trait anhedonia in a non-clinical sample (i.e. college students) [30, 41]. The RSAS and RPAS have been used principally to assess schizotypy rather than anhedonia per se, and most previous studies have not included a large number of participants [30, 40, 41]. Thus, questions remain regarding the applicability of the RSAS and the RPAS, particularly with Chinese respondents.

**Table 1:** Recent studies on the Revised Social Anhedonia Scale (RSAS) and Revised Physical Anhedonia Scale (RPAS)

	Study author (year)	Participants	Topic	Reliability and validity
America	Chapman et al. (1976) [1]	505 normal adults & male schizophrenics	The first version of two Chapman scales-Physical Anhedonia Scale (PAS) and Social Anhedonia Scale (SAS). Both scales can measure anhedonia effectively among healthy adults and schizophrenics, but the PAS would appear more likely to reflect a biological defect schizophrenic anhedonia.	Cronbach α: PAS: 0.74 (male); 0.66 (female) SAS:0.85 (male); 0.82 (female) The two scales correlated 0.60 for males and 0.51 for females.
	Blanchard JJ et al. (1998) [35]	37 schizophrenia outpatients & 15 controls	Used PAS and SAS as a tool to measure anhedonia and to examine the relationship between anhedonia and the trait dimensions of positive affect (PA) and negative affect (NA) in schizophrenia. The first applied research of PAS and SAS in schizophrenia.	RSAS: Cronbach α: 0.84 Test-retest: Schizophrenia:0.79; Control:0.82 RPAS Cronbach α: 0.86 Test-retest: Schizophrenia:0.74; Control:0.86
	Kwapil et al. (2008) [46]	6137 college undergraduates	Reported the dimensional structure of the Wisconsin Schizotypy Scales (WSS), which including the RPAS, RSAS and other two scales. Physical anhedonia (RPAS) and social anhedonia (RSAS) were recognized negative schizotypy factors of the WSS.	Two-factor model of WSS: RMSEA:0.053; CFI:0.98; GFI:0.98
	Leventhal et al. (2006) [52]	157 college students	To measure hedonic capacity in Depression. The RPAS (anhedonia) was positively related to depression.	Cronbach α:0.93 (RPAS) RPAS score was correlated with BDI (*r* = 0.16, *p* < o.o5) Three-factor model: RMSEA:0.07; CFI:0.97; TLI:0.94
	Reise et al. (2011) [42]	2227 18-year-olds	Item response theory model in the RSAS: the RSAS responses cannot be modeled accurately by either unidimensional or bifactor IRT models. Although the scores on the RSAS are meaningful and valuable in this study, neither of the model could reflect the true relation between the items and the target latent trait.	Cronbach α: 0.84 (RSAS) Unidimensional model: RMSEA = 0.05; CFI = 0.88 Bifactor model: RMSEA = 0.03; CFI = 0.97
	Cicero et al. (2016) [45]	584 undergraduates (EFA) 932 undergraduates (CFA)	To examine factor structure of the RSAS and examine whether the RSAS has discriminant validity from social anxiety.	Two-factor model: RMSEA = 0.03, CFI =0.93
Spain	Fonseca-Pedrero et al. (2009) [29]	728 Spanish university students	The psychometric properties of the RPAS and the RSAS in Spanish population. Both scales presented an essentially unidimensional solution.	RSAS: Cronbach α:0.95; Unidimensional model: RMSEA =0.067; CFI =0.92 RPAS:Cronbach α:0.92; Unidimensional model: RMSEA =0.028; CFI =0.99
Germany	Bailer et al. (2004) [37]	83 schizophrenic patients and 83 normal controls	To test reliability and validity properties of German versions of the WSS. The satisfactory internal consistency and high retest-reliability of the RPAS and RSAS were shown in both groups	Cronbach α: 0.73–0.87 Test-retest: 0.54–0.87
French	Loas (1993) [36]	61 normal subjects, 61 major depressive disorder	This work presents the validation of the French version of the Physical Anhedonia Scale (PAS). The scale’s validity, fidelity and reliability were studied in two groups	Cronbach α: 0.71–0.83(PAS) PAS scores were correlated with Fawcett–Clark Pleasure Capacity Scale (FCPS) (*r* = − 0.53, *p* < o.o1)
China	Chan et al. (2012) [30]	887 college students	The RPAS and RSAS were used separately to measure trait ahnedonia in a non-clinical sample.	Cronbach α: RSAS: 0.85; RPAS:0.86
	Chan et al. (2015) [41]	1724 young adults	The factor structure and the measurement invariance across time of the WSS. Physical anhedonia (RPAS) and social anhedonia (RSAS) were recognized as negative schizotypy factors. Measurement invariance of the WSS across time was supported.	Cronbach α:(time1, time2, time1_parceled_ and time2_parceled_) RSAS:0.82;0.85;0.81;0.84 RPAS:0.83;0.87;0.83;0.87 Two-factor model: RMSEA:0.049; CFI:0.98; TLI:0.97
	Chan et al. (2016) [40]	196 schizophrenia patients, 197 non-psychotic first-degree relatives, 1724 non-clinical young adults	The structural invariance across goups of the WSS. Two-factor model was confirmed in three groups. Physical anhedonia (RPAS) and social anhedonia (RSAS) were recognized as negative schizotypy factors.	schizophrenia patients: Cronbach α: 0.83–0.93 RMSEA:0.079; CFI:0.96; TLI:0.94 First-degree relatives: Cronbach α: 0.82–0.94 RMSEA:0.09; CFI:0.95; TLI:0.93 College students: Cronbach α: 0.75–0.89 RMSEA:0.059; CFI:0.97; TLI:0.96
Turkey	Cihan et al. (2015) [31]	266 s-grade university students	Confirmatory factor analysis was performed to test schizotypy dimensions. The Cronbach’s alpha, test–retest reliability and congruent validity of SAS were calculated.	Cronbach α: 0.84 (SAS) Test-retest: 0.76 Two-factor model (positive–negative schizotypy): RMSEA:0.07; CFI:0.96; GFI:0.94

Although the factor structures of Chapman’s physical and social anhedonia scales have long been considered unidimensional, as was the intention when they were developed [1, 42], and that single dimension has been classified as a negative dimension of schizotypy in the context of Meehl’s model [29], emerging evidence indicates that the RPAS and RSAS may have more complicated structures [43, 44]. The structure of the RPAS alone has not yet been reported, and that of the RSAS remains controversial, with studies reporting one [29, 43], two [45], and four [44] factor model fits, depending on language and subject sample. The two factors of the 2-factor RSAS model were defined as social apathy/aversion and social withdrawal, which are associated with the symptoms of the schizophrenia [44]. When the RSAS was loaded in both positive and negative WSS factors, model fitness outcomes were better than when RSAS was loaded in a purely negative factor [40, 46–48]. Although the two components of anhedonia (anticipatory and consummatory) have been distinguished in behavioral and psychometric studies [49], it remains to be clarified whether the anticipatory and consummatory anhedonia components exist in both the physical and social fields, and whether there is a hierarchical relationship between them [50]. Besides, although the RPAS and RSAS have been used together across different cultures [29, 30, 51], they have never been recognized as a combined unified scale (referred to as the RPAS&RSAS from here forward), and the factor structure of the RPAS&RSAS remains unrevealed.

The RPAS and RSAS, generally considered to be traditional anhedonia scales, were designed for patients with schizophrenia [1] and have been used extensively to assess anhedonia in schizophrenia studies [52]. Their appropriateness in other populations is unclear and has not been validated empirically [16]. Thus, there is a need to investigate their reliability and validity in other patient populations and non-clinical samples. The aim of this study was to analyze the individual structures of the RPAS and RSAS separately and the RPAS&RSAS as a combined unified scale to explore the underlying construct of anhedonia both in clinical patient and undergraduate samples. Firstly, we used Exploratory Factor Analysis (EFA) to explore the structure of the RPAS and RSAS in undergraduate samples. Secondly, we used confirmatory Factor Analysis (CFA) to confirm the structures of the RPAS and RSAS both in clinical patient and undergraduate samples. Third, we analyzed the structure of the unified RPAS&RSAS for how it fits into a potential hierarchical model in clinical and undergraduate samples. The results obtained may be used to broaden the application of the RPAS and RSAS as measures of anhedonia, including in the context of various neuropsychiatric conditions, including anxiety and depression.

## Methods

### Participants

The sample size was calculated using the criteria of subject-to-item ratio of 10:1 or more [53], which giving a 1010 required sample size for EFA or CFA analysis. Based on the required sample size, this study recruited undergraduate sample from two Chinese universities in Hunan Province and they finished the scales in classroom. A total of 3537 university students completed the survey and 102 (2.8%) subjects were excluded. So 3435 subjects with full data were left, including 1633 males and 1802 females, with mean ages ± standard deviations of 18.37 ± 0.23 years and 18.11 ± 0.28 years, respectively. We assessed the mental disorders, a family history of any mental disorder, and physical disorders by self-reporting using a self-made questionnaire. The exclusion criteria were: (1) history of mental disorder, (2) family history of mental disorder (3) history of neurological disorder, (4) intellectual disability. To estimate test-retest reliability, we randomly selected 10 classes of students as a subgroup who had been completed the RPAS and RSAS twice with a one-month interval. Finally 223 students completed the retest questionnaire. The students did not responded anonymously and the tests were unpaid, and all the students volunteered to complete the test. If any student didn’t want to participate, they could refuse to fill it out or drop out at any time.

The criteria of sample size is at least reach minimum sample size of 200 [54], after that, we try to meet the criteria of subject-to-item ratio of 10:1 [53]. In this study, the clinical samples reached the previous criteria, while the college student samples met both criteria. For the clinical sample, 348 clinical patients who had been referred for assessment and treatment in the psychological clinic of Second Xiangya Hospital were recruited. As subject-to-item ratio of 10:1 was not satisfied in the patient sample, the minimum sample size of 200 which was also considered in student sample was used in clinical patients. Finally, a total of 294 patients finished the questions, including 146 men (50%) and 148 women (50%), aged 16 to 37 years old (Mean = 24; standard deviation (SD) = 15.9). The clinical sample only included psychiatric disorders which were ever reported to be correlated to anhedonia, such as major depressive disorder, obsessive-compulsive disorder, schizophrenia and the related personality disorder (depressive personality disorder, obsessive-compulsive personality disorder and schizotypal personality disorder). There was a significant age difference between the undergraduate sample and the clinical sample, the clinical sample was significantly older than the undergraduate sample (t = 5.831, *p* < 0.001), but no significant gender difference was found between the two samples (χ^2^ = 0.841, df = 1, *p* = 0.361). The score of the Beck Depression Inventory (BDI), the Beck Anxiety Inventory (BAI) and the Childhood Trauma Questionnaire (CTQ) of clinical patients are significantly higher than those of undergraduate sample (*p* < 0.001). Moreover, the PRAS, the RSAS and the RPAS&RSAS score of clinical patients are also significant higher than undergraduate sample(*p* < 0.001). The socio-demographic of the two samples in detail are presented in Table 2.

**Table 2:** Socio-demographic characteristics of samples

Characteristic description		Undergraduates Sample (*n* = 3435)	Clinical Sample (*n* = 294)	Chi-Square/t	*p*	Cohen’s d
Gender, n	Male/female	1633/1802	146/148	0.841	0.361	–
Age, Mean ± SD	Years	18.73 ± 0.78	24.02 ± 7.52	5.831	< 0.001	–
Scale, Mean ± SD	BDI	6.51 ± 6.91	12.44 ± 8.88	−11.22	< 0.001	0.73
	BAI	26.71 ± 6.76	30.74 ± 7.95	−7.78	< 0.001	0.54
	CTQ	47.19 ± 7.95	49.94 ± 14.30	−4.33	< 0.001	0.22
	RPAS	17.33 ± 7.61	20.32 ± 9.31	−6.58	< 0.001	0.34
	RSAS	9.06 ± 5.70	15.41 ± 7.96	−18.53	< 0.001	0.89
	RPAS&RSAS	22.46 ± 11.50	35.69 ± 15.31	−13.40	< 0.001	0.95

*BDI* beck depression inventory, *BAI* beck anxiety inventory, *CTQ*: childhood trauma questionnaire, *RPAS* revised physical anhedonia scale, *RSAS* revised social anhedonia scale

Participants were told that the information in these scales would not be disclosed to anyone outside of the research team and written informed consent forms were completed by all participants. This study was approved by the ethics committee of Second Xiangya Hospital, Central South University.

### Materials

#### Revised physical Anhedonia scale (RPAS) and social Anhedonia scales (RSAS)

Chinese versions of the RPAS and RSAS that were translated by linguists and psychologists from English into Chinese were used [34]. The RPAS focuses on typically pleasurable physical stimuli (e.g. food), whereas the RSAS assesses anhedonia related social stimuli and connection with others. The RPAS and RSAS contain 61 and 40 true-false items, respectively. The items are scored relative to standard answers. According to the standard answer, items that need reverse scored are ‘False’, and items that do not need reverse scored are ‘True’. If the response to an item matches the item’s standard answer, it is scored as a “1”; otherwise, it is scored as a “0”. Higher scores are indicative of more severe anhedonia and elevated risk of mental disorders. Both the original English RPAS/RSAS (α = 0.74/0.85) and the Chinese RPAS/RSAS (Cronbach’s α = 0.75/0.94) have good internal consistencies (see Table 1).

#### Beck depression inventory (BDI)

The BDI is a multiple-choice self-reporting 21-item scale [55] used primarily to assess the presence and severity of depressive symptoms in the prior 2 weeks in clinical and non-clinical populations (e.g. guilty feelings; loss of pleasure). Each question is answered on a 0–3-point scale of intensity. The BDI total score range is from 0 to 63 points, with higher scores indicating more severe symptoms. In this study, the Cronbach’s α of BDI was 0.85 in undergraduate sample and 0.84 in clinical sample.

#### Beck anxiety inventory (BAI)

The BAI includes 21 items [56] that assess the degree to which subjects are disturbed by various anxiety symptoms (e.g. Unable to relax; Nervous). Each item is scored on a 4-point scale, ranging from 1 (not at all) to 4 (severely). The total score ranges from 21 to 84. In this study, the Cronbach’s α of BAI was 0.90 in undergraduate sample and 0.83 in clinical sample.

#### Childhood trauma questionnaire (CTQ)

The CTQ is a 28-item [57] retrospective self-report questionnaire to assess childhood trauma experience before the age of 16. It has five subscales: emotional neglect (e.g., “felt loved,”), physical neglect (e.g., “was taken to the doctor,”), emotional abuse (e.g., “felt emotional abused”), sexual abuse (e.g., “was molested,”) and physical abuse (e.g., “was hit so hard by family”). Five items each assess all five types of maltreatment, another 3 items was taken as validity evaluation (e.g., “perfect childhood”). Each item is scored on a 5-point scale. Each subscale was varied from 5 to 25 scores and the total score was in the range from 25 to 125 score. In this study, the Cronbach’s α of CTQ was 0.72 in undergraduate sample and 0.68 in clinical sample.

#### Data analysis

IBM SPSS Statistics 20.0 was used for descriptive statistics and M-plus 7.11 software was used for factor analysis (EFA and CFA). Data from participants with missing data and participants that met the exclusion criteria were excluded. Mean descriptive statistic values are reported with 95% confidence intervals (CIs). Cronbach’s α values were calculated to evaluate reliability (i.e. internal consistency). A minimum standard of 0.70 is set for Cronbach’s α coefficients, but an α of 0.60 is also considered acceptable. Test-retest reliability was assessed with Pearson correlation analyses. Some items (physical, 1/4/9/40/53, and social 33) with low relevance or inappropriate meaning were excluded from the Chinese RPAS and RSAS to make them more suitable for Chinese youth. The excluded items had Pearson r values < 0.100, indicating very weak associations with pleasure capacity in Chinese youth. Additionally, four of the excluded physical items (1, 9, 40, and 53) address sexual issues; these items can be excluded when applied to teenagers with limited sexual experience .

In the undergraduate sample, we split the collected statistics in half randomly. There is no difference in age and gender between two samples. First, the EFA was conducted on a randomly split-half of the whole sample (*n* = 1770). EFA was used to identify the best fitting factor model of the RPAS and RSAS, in the present sample. Subsequently, we used a random split-half (*n* = 1769) of the sample to run CFA. The CFA tested the fit of the model that was generated from our EFA. Finally, utilizing the full sample we conducted CFA to estimate the factor structure of the simplified unified RPAS&RSAS. Besides, we also conducted the CFA to estimate the structure of the RPAS, the RSAS and the unified RPAS&RSAS among clinical patients.

In the EFA, categorical variables were analyzed with the mean and variance adjusted (WLSMV) estimator, the most common and effective analysis method for categorical variables [58], in the M-plus program. Parceling was not performed in the EFA because the scale structures were unknown.

In preparation for CFA, items were parceled into individual factors according to the internal consistency approach for multidimensional scales to simplify the unified RPAS&RSAS. A random algorithm was used in the parceling for its convenience and validity. For four-item parceled items, mean parcel scores were used as final scores, thereby changing categorical variables into quantitative variables. CFA of the simplified unified RPAS&RSAS was thus conducted with a maximum likelihood (ML) estimator for the first-order (physical anhedonia and social anhedonia) and second-order (anticipatory physical/social anhedonia and consummatory physical/social anhedonia) models. The Tucker-Lewis index (TLI), comparative fit index (CFI), standard root mean square residuals (SRMR), and root mean square error of approximation (RMSEA) methods were used to determine goodness of fit [59]. The criteria for accepting the model were: TLI ≥ 0.900, CFI ≥ 0.900, SRMR ≤0.080, and RMSEA ≤0.080 [60, 61].

## Results

### Descriptive statistics

In the undergraduate sample, 32 students were removed from the sample due to being outliers, total scores for the uniformed RPAS&RSAS ranged from 2 to 61 (Mean = 22.46; SD = 11.50). The skewness for each item ranged from .04 to 3.65, kurtosis ranged from .01 to 3.66 (Table S1 in supplementary material).

In clinical sample, total scores for the uniformed RPAS&RSAS ranged from 7 to 80 (Mean = 35.69; SD = 15.31). The skewness for each item ranged from .02 to 2.65, kurtosis ranged from .01 to 2.83 (Table S2 in supplementary material).

### Reliability: internal consistency and test-retest reliability

The Cronbach’s α coefficient of the RPAS&RSAS were 0.884 and 0.672 in the undergraduates and the clinical patients respectively, which indicated good reliability with respect to internal consistency. The Pearson’s *r* values obtained for the RPAS&RSAS 0.644 (*p* < 0.001) in a subsample of 223 participants, indicating good test-retest reliability.

### Construct validity: RPAS/RSAS factor analysis

After the elimination of six items for culture inappropriateness or poor relevance (see Methods), EFA of the RPAS (56 items) and RSAS (39 items), involving half of the participants, was conducted with a WLSMV estimator. The model fit indexes obtained for the RPAS and RSAS are presented in Table 3 and Table 4, respectively. As illustrated in Table 3, only the 4-factor model meet the criteria for accepting the model (CFI = 0.924, TLI = 0.912, SRMR = 0.053, RMSEA = 0.024). Thus, the 4-factor model was the best fitting factor structure of the RPAS. The RSAS fit well with a 2-factor structure model (CFI = 0.941, TLI = 0.934, SRMR = 0.063, RMSEA = 0.028) as shown in Table 4. The factor loadings of each item are reported in Table S3 and Table S4 in the supplementary material.

**Table 3:** The fitness indicators in the EFA of the models of the RPAS

Model	Chi-Square	df	CFI	TLI	SRMR	RMSEA(90%CI)
1-factor	7866.714	1430	0.625	0.611	0.133	0.051 (0.050, 0.052)
2-factor	4036.320	1367	0.845	0.833	0.073	0.034 (0.032, 0.035)
3-factor	3226.631	1375	0.897	0.884	0.060	0.028 (0.027, 0.029)
4-factor (modified 2-factor)	2567.392	1271	0.924	0.912	0.053	0.024 (0.023, 0.026)

*RPAS* revised physical anhedonia scale, *df* degree of freedom, *CFI* comparative fit index, *TLI* tucker lewis index, *SRMR* standard root mean square residuals, *RMSEA* root mean square error of approximation, *90% CI* 90% confidence Interval

**Table 4:** The fitness indicators in the EFA of the models of the RSAS

Model	Chi-Square	df	CFI	TLI	SRMR	RMSEA(90% CI)
1-factor	4436.681	702	0.756	0.742	0.130	0.056 (0.054, 0.057)
2-factor	1569.098	664	0.941	0.934	0.063	0.028 (0.026, 0.030)

*RSAS* revised social anhedonia scale

In the RPAS, the physical anhedonia items segregated into four factors as follows: Factor 1 contains items 5, 6, 8, 10, 11, 12, 13, 14, 16, 17, 18, 20, 22, 23, 26, 27, 28, 29, 32, 33, 43, 44, 48, 50, 51, 52, 55, 56, and 57; Factor 2 contains items_2, 3, 19, 21, 24, 25, 30, 31, 34, 35, 36, 37, 39, 41, 45, 46, 47, 49, 54, 58, 59, 60, and 61; Factor 3 contains items 7 and 15; and Factor 4 contains items 38 and 42. However, as can be seen in Table S4, Factor 3 and Factor 4 each have only two items, and all four of these items had relatively high loadings (loadings > 0.350 [62]) in the first two factors (item 15 loaded 0.448 in Factor 1; item 7, 38, 42 loaded 0.389, 0.471 and 0.363 in Factor 2 respectively). Thus, we spread these four items into the first two factors according to loading parameters. Accordingly, item 15 was placed with Factor 1 and items 7, 38, and 42 were placed with Factor 2.

The RPAS items gathered in Factor 1 (P1) were related to consummatory physical pleasure whereas the RPAS items gathered in Factor 2 (P2) were related to anticipatory physical pleasure. Regarding items gathered in Factors 3 and 4, the content of item 15 (There aren’t many things I really like to do) associated well with “liking”, thus relating it to consummatory physical anhedonia (Factor 1). In contrast, the content of items 7 (The taste of food has always been important tome), 38 (The beautiful scenery can make me feel delighted). and 42 (I seldom have the idea of singing in the bath) associated well with “wanting”, thus relating them to anticipatory physical anhedonia (Factor 2).

The RSAS items gathered in Factor 1 (S1)—that is, items 1, 2, 3, 6, 10, 13, 14, 17, 21, 22, 23, 26, 27, 28, 29, 32, 34, 35, 37, 38, 39, and 40—were related to the consummatory social pleasure. The RSAS items gathered in Factor 2 (S2)—that is, items 4, 5, 7, 8, 9, 11, 12, 15, 16, 18, 19, 20, 24, 25, 30, 31, and 36—were related to anticipatory social pleasure.

For CFA of two scales in undergraduate samples, we parceled randomized four items and simplified the scales because the RPAS/RSAS items seemed excessive and scattered. There were 14 parcels in the RPAS (7 in P1 and 7 in P2) and 9 parcels in the RSAS (5 in S1 and 4 in S2). As is shown in Table 5, fit index values supported a good fit with a 2-factor model for the RPAS (CFI = 0.947, TLI = 0.936, SRMR = 0.039, RMSEA = 0.052) and a good fit with a 2-factor model for the RSAS (CFI = 0.967, TLI = 0.957, SRMR = 0.037, RMSEA = 0.048) in CFA of the undergraduates, consistent with our EFA results. In the clinical sample, the results of CFA corresponded with the non-clinical sample’s and supported 2-factor models for both the RPAS and the RSAS (RPAS: CFI = 0.947, TLI = 0.932, SRMR = 0.044, RMSEA = 0.052; RSAS: CFI =0.977, TLI = 0.968, SRMR = 0.041, RMSEA = 0.061).

**Table 5:** The fitness indicators the CFA and the models of the RPAS and the RSAS

	Chi-Square	df	CFI	TLI	SRMR	RMSEA(90%CI)
Undergraduates						
RPAS	445.376	76	0.947	0.936	0.039	0.052 (0.048,0.057)
RSAS	171.468	34	0.967	0.957	0.037	0.048 (0.041,0.055)
Clinical patients						
RPAS	133.039	76	0.947	0.932	0.044	0.051 (0.036,0.065)
RSAS	53.231	26	0.977	0.968	0.041	0.061 (0.037,0.084)

### Factor analysis of the RPAS&RSAS

**Fig. 1:**
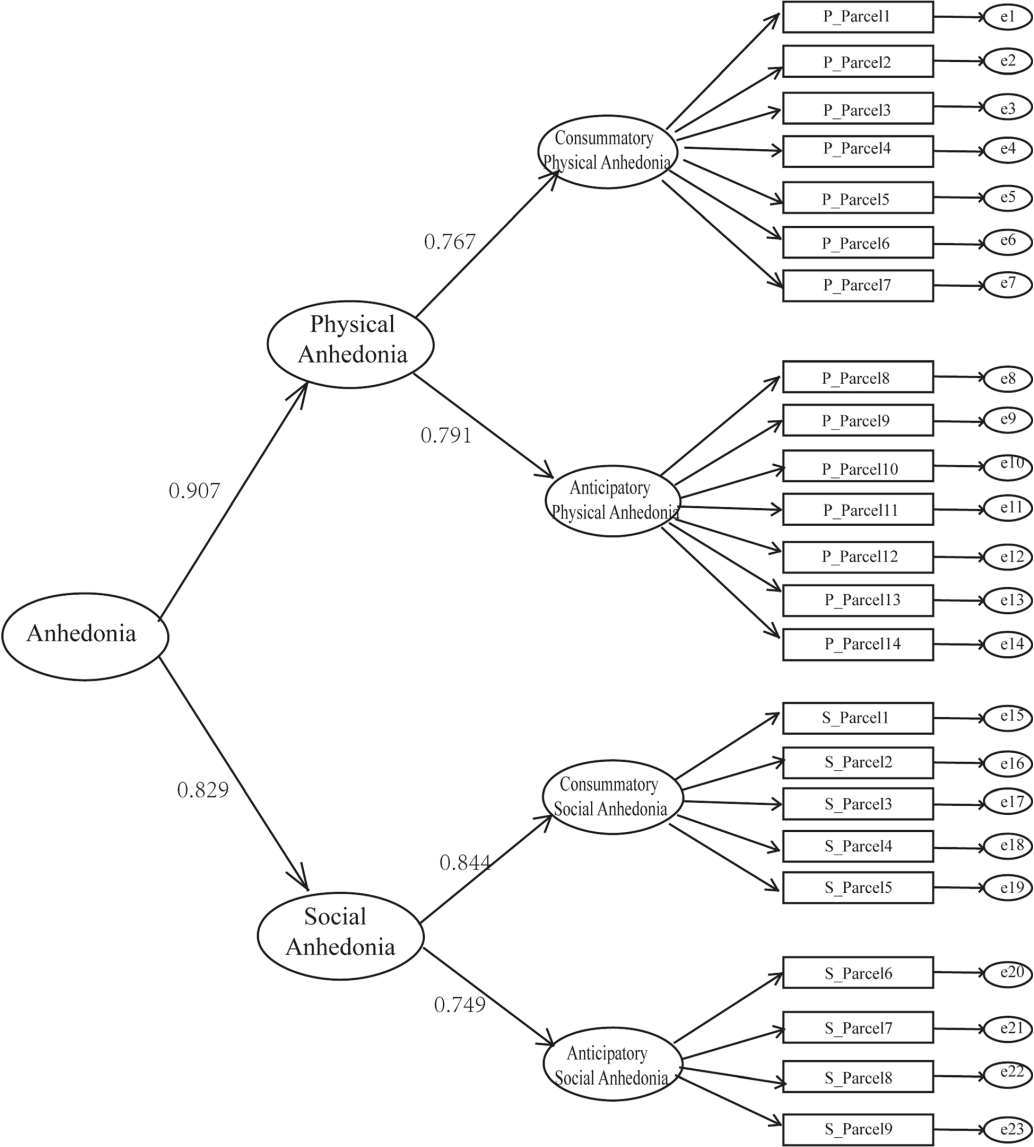
Second-order model of the Revised Physical and Social Anhedonia Scales (RPAS and RSAS) and the underlying construct of anhedonia

Based on the good fits of the 2-factor models for the two individual scales and good first-order model fitness results for the RPAS&RSAS (Table 6), CFA confirmed a novel second-order model for the RPAS&RSAS as a unified scale. The second-order model were good fitted in clinical sample: CFI = 0.922, TLI = 0.911, SRMR = 0.078, RMSEA = 0.052). But in undergraduate sample the second-order model was good fitted overall (CFI = 0.901, TLI = 0.899, SRMR = 0.086, RMSEA = 0.055) except that SRMR was slightly missed the margin of criteria for acceptable fit. The model fit indexes supported a simpler construct of anhedonia, as shown in Fig. 1. The results supported that with a lower number of degrees of freedom and a more simple structure, the second-order model showed unique advantage in hierarchy to support the perspective of multiple dimensions in anhedonia, which combines observable behavioral symptoms and underlying biological mechanisms.

**Table 6:** The fitness indicators in the CFA of the models of the RPAS&RSAS

	Chi-Square	df	CFI	TLI	SRMR	RMSEA(90%CI)
Undergraduates						
First-order model	1628.053	224	0.941	0.934	0.038	0.042 (0.040, 0.044)
Second-order model	2592.801	225	0.901	0.899	0.086	0.055 (0.053, 0.056)
Clinical patients						
First-order model	362.611	224	0.936	0.928	0.033	0.047 (0.038, 0.056)
Second-order model	390.599	222	0.922	0.911	0.078	0.052 (0.044, 0.061)

## Discussion

The current study examined the psychometric properties of a unified RPAS&RSAS as a distinct anhedonia assessment tool in a large sample of young Chinese adults and clinical patients for the first time. The results not only showed that good reliabilities and validities of RPAS&RSAS, but also supported an underlying hierarchical structure of anhedonia. Specifically, we accepted a simple and accurate second-order model of the RPAS&RSAS, which was confirmed to have a good fit in clinical patients, supporting the robust construct validity of the combined unified scale. As anhedonia is composed of consummatory anhedonia and anticipatory anhedonia from the reward processing models in the neurobiology, the construct of RPAS&RSAS (the “gold standard” for measuring hedonic capacity) is of great possibility to be consistent with the components of anhedonia.

Initially, anhedonia was considered as a psychopathological symptom among clinical patients. According to external behavior of patients, phenomenological researches suggested anhedonia had two major components, including physical anhedonia and social anhedonia [29]. In the past two decades, with further researches on the genetic, phenomenological, and molecular mechanisms of it, anhedonia was more and more considered as a multidimentional concept that encompasses physical/social dimension, consummatory/anticipatory dimension, and so on [30]. The current study combined the RPAS and RSAS as a whole to assess its psychometric properties in both clinical and non-clinical samples, and further explored the multiple conceptual dimensions of anhedonia contained in this scale. The results showed that it was feasible and suitable to use a unified RPAS&RSAS instrument as a novel distinct instrument, in which the acceptable Cronbach’s α values supported good internal consistency for anhedonia (physical and social), and the high RPAS&RSAS test-retest reliability coefficient indicated the temporal stability of anhedonia. Additionally, a good fitting yielded by the second-order model in this study provided new evidence to the multi-dimensionality of anhedonia. It should be noted that a previous study showed that RPAS and RSAS may have high degree of cultural bias demonstrated from questions [16]. Further researches are needed to assess the measurement invariance of unified RPAS&RSAS cross cultures.

With respect to the factorial validity, the RSAS was found to have a 2-factor structure both in undergraduates and clinical patients in this study, with the two components being defined as consummatory and anticipatory social anhedonia. This 2-factor model is consistent with Cicero’s et al.’s study [44], with only items 24 and 25 having different factor designation relative to the EFA results. Cicero defined their two RSAS factors as social apathy/aversion and social withdrawal based on an emotion and behavior perspective, and in the context of potential symptoms of schizophrenia [44]. According to the recent research progress on anhedonia, we proposed that the two factors explored by both studies may reflect the common underlying mechanisms of anhedonia prior described in numerous previous literature: the anticipatory and consummatory anhedonia [63]. Specifically, anticipatory social anhedonia, or social withdrawal defined by Cicero, is characterized by a loss of the motivation to connect to others; while consummatory social anhedonia, or social apathy/aversion, is characterized by a lack of emotional connection or involvement in social activities [7]. The terms consummatory social anhedonia and anticipatory social anhedonia can be related to potential biological mechanisms whereas the terms social withdrawal and social apathy are derived from schizotypy symptoms. It is hoped that the context and links made in this study may promote application of the RSAS for assessment of a broader range of mental disorders (beyond the schizophrenia spectrum) in the future [7, 13, 49].

Similar to our findings with the RSAS, we also obtained a 2-factor structure for the RPAS: consummatory physical anhedonia and anticipatory physical anhedonia. We excluded items 1, 4, 9, 40, and 53 from the RPAS due to their low relevance, consistent with the previous study [54]. The RPAS has long been assigned to the negative schizotypy dimension in the WSS, in which its unidimensionality has been taken for granted [1, 64]. However, animal behavioral experiments in depression [65] and schizophrenia [66, 67] models suggested that physical anhedonia has more than one dimension. For example, the sucrose preference test relies on an affinity for a physical stimulus (consummatory pleasure) whereas the forced swimming test relates to “wanting” (anticipatory pleasure) [16]. Elucidation of the anhedonic capacity concept [7], including consummatory and anticipatory components, in the context of neurobiological arguments underscores an inherent complexity of the RPAS construct. Meanwhile, the potential utility for using the RPAS to assess anhedonia generally over the lifespan also points to its potential multi-dimensionality [52]. Finally, data from other measures support a psychometric multidimensionality of physical anhedonia. For example, some items in the Temporal Experience of Pleasure Scale involve the pleasure of tasting food and these aspects of pleasure were represented in the consummatory and anticipatory components of the RPAS [49], as indicated in our 2-factor model.

It is worthy to note that our study not only confirmed the 2-factor model including consummatory and anticipatory components in both RPAS and RSAS, but also proved a simple and accurate second-order model of the RPAS&RSAS to further support the robust construct validity of the combined unified scale. In addition, that hierarchical model was confirmed to have a good fit in both the sample of undergraduate students and clinical patients. In fact, the notion of anhedonia as encompassing social and physical aspects was developed in the 1970s. There is a long history and broad acceptance of viewing mental disorders in relation to physical sensation and social function, and this perspective is well represented in diagnostic criteria. These bases are also amenable to experimental exploration of the nature of anhedonia in animal experiments [68, 69]. The recent theory of temporal components of anhedonia, namely anticipatory and consummatory anhedonia [7], were also supported by our hierarchical analysis of the concept of anhedonia.

In our model shown in Fig. 1, anhedonia is divided firstly into physical and social anhedonia and secondly each aspect of anhedonia is divided into anticipatory and consummatory components. The unified RPAS&RSAS, separated from the WSS, has proven to be a scale of simple construction that integrates multiple levels of information related to anhedonia, which makes it a convenient and useful tool for researchers and clinical psychologists. Hedonic capacity has been related to genetics, neurochemical disorders, and specific brain regions [2, 50]. From the transdiagnostic perspective, anhedonia is considered as the emotion and reward processing deficiency and reflects an endophenotype (i.e. an intermediate phenotype of trait that is expressed along a spectrum), which can be a component of multiple mental disorders, including schizophrenia and depression [12, 70]. Additionally, those neuropsychiatric deficit endophenotypes have been linked to genetic signatures and molecular mechanisms [71–73]. Thus, the second-order structure found for the RPAS&RSAS may help provide insight into the integration of multiple levels of information, including genetic, endophenotype, and symptom expression, in various mental disorders from the perspective of an anhedonia measure.

According to the previous literatures, anhedonia is a prominent symptom of several neuropsychiatric disorders, especially in major depressive disorder (MDD) and schizophrenia [13, 18]. Anhedonia is characterized as “loss of interest or pleasure in daily life” and recognized as one of two essential features of MDD [74, 75]. Meanwhile, anhedonia is also the most common negative symptom of schizophrenia, manifested as apathy or indifference [18]. According to the National Institute of Mental Health (NIMH) Research Domain Criteria (RDoC) project, researchers trying to use the basic behavioral dimension of functioning to identify anhedonia in a transdiagnostic approach. Thus, a suitable tool is needed to measure the anhedonia comprehensively. Based on the latest studies, anhedonia is a multidimensional construct, such as physical/social, anticipatory/consummatory or motivation/experience dimension. In clinical observation, patients with mental illness show different patterns of composition in the subcomponent of anhedonia. For instance, patients with schizophrenia reported more remarked social anhedonia than physical anhedonia [76, 77]. However, in patients with major depressive disorder, both significant physical anhedonia and social anhedonia were observed [78, 79]. Similarly, a number of studies showed that patients with different diagnoses had different impairments in anticipatory and consummatory aspects. A recent meta-analysis of anticipatory and consummatory pleasure in schizophrenia reported that anticipatory pleasure may be significantly impaired in patients compared to the consummatory pleasure [80]. An ALE meta-analysis also revealed that consummatory and anticipatory were associated with different neural mechanisms of anhedonia in MDD and schizophrenia [4]. Hence, exploring the construct of anhedonia and distinguishing the different subcomponents of anhedonia are essential for mental illnesses. However, there is no qualified assessment both covering the classification of physical and social anhedonia, as well as anticipatory and consummatory anhedonia, which is in need for exploring the anhedonia in various kinds of psychiatric disorders. Thus, this study confirmed the good reliabilities and validities of RPAS&RSAS as a unified measurement and revealed a second-order model in both healthy and clinical samples, which provides new possibilities for measuring anhedonia in various mental disorders and help to promote more precise treatment approaches.

The RSAS and RPAS were comprehensive measurement tools for assessing anhedonia. Compare to rating scale, the RPAS&RSAS was written in simple language with a yes/no response format, which makes it easy to understand and patients spend less time to finish [29]. Since its comprehensive and easy to understand, the RSAS and RPAS has been widely used in clinical patients. In this study, it only took about 10 min for the clinical patients to complete the combining scale.

Although the results of the present study demonstrated a stable second-order model and appropriate reliability scores for the Chinese version of the unified RPAS&RSAS, several limitations should to be acknowledged. Firstly, reliance on an undergraduate student sample may limit the generalizability of our findings to individuals in other age bands. Secondly, our results are based on a cross-sectional study, which did not take into consideration changes that may occur over time within individuals. Further longitudinal studies should be conducted to investigate the psychometric properties of the unified RPAS&RSAS in more age group.

## Conclusion

In summary, the study explored the psychometric properties of the RPAS, the RSAS and the unified RPAS&RSAS in the undergraduates and clinical patients. A second-order model of the RPAS&RSAS was brought out for the first time and was selected over a first-order model for its ability to optimize simplicity and accuracy. The novel construct for anhedonia was further confirmed both in healthy undergraduate sample and clinical sample. Our study may promote the understanding of the multiple dimensions in anhedonia and broaden the application of the unified RPAS&RSAS as an individual measurement in various psychiatric disorders.

## Supplementary information

**Additional file 1.**

## Data Availability

The datasets generated and analysed during the current study are not publicly available due to no permission from participants to share anonymized participant data publicly but are available from the corresponding author on reasonable request.

## Abbreviations

RPAS: Revised physical anhedonia scale
RSAS: Revised social anhedonia scale
RPAS&RSAS: Unified, simplified RPAS and RSAS
EFA: Exploratory factor analysis
CFA: Confirmatory factor analysis
WSS: Wisconsin Schizotypy Scales
