# Supplementary material for: A novel construct of anhedonia revealed in a Chinese sample via the Revised Physical and Social Anhedonia Scales
Source: BMC Psychiatry. 2020 Nov 9;20:529. doi: 10.1186/s12888-020-02900-w (PMC7650163; doi:10.1186/s12888-020-02900-w)
Supplement: Supplementary file 1 — Additional file 1. [file 12888_2020_2900_MOESM1_ESM.docx]

**A novel construct of anhedonia revealed in a Chinese sample via the revised physical and social Anhedonia scales**

Qiongqiong Wu, Jiayue He, Shulin Fang, Panwen Zhang, Xingwei Luo, Jianghua Zhang, Yan Xiong, Fusheng Luo, Xiaosheng Wang**,** Shuqiao Yao, Xiang Wang

**Supplementary Material**

**Table S1. The Descriptive results of the Revised Physical and Social Anhedonia Scale in undergraduate sample**

**Table S2. The Descriptive results of the Revised Physical and Social Anhedonia Scale in clinical sample**

**Table S3. Factor Loadings for the Item-Level in EFAs of the RPAS and RSAS**

**Table S4. Factor Loading of the Revised Physical Anhedonia Scale in EFA**

**Table S1. The Descriptive results of the Revised Physical and Social Anhedonia Scale in undergraduate sample**

| Item | Mean | SD | Skew | Kurt | Item | Mean | SD | Skew | Kurt |
| --- | --- | --- | --- | --- | --- | --- | --- | --- | --- |
| physical_1 | 0.45 | 0.50 | 0.18 | -1.96 | physical_52 | 0.50 | 0.50 | 0.01 | -2.00 |
| physical_2 | 0.47 | 0.50 | 0.13 | -1.98 | physical_53 | 0.73 | 0.44 | -1.05 | -0.88 |
| physical_3 | 0.29 | 0.45 | 0.92 | -1.15 | physical_54 | 0.08 | 0.26 | 3.21 | 3.350 |
| physical_4 | 0.50 | 0.50 | 0.04 | -2.00 | physical_55 | 0.29 | 0.45 | 0.92 | -1.153 |
| physical_5 | 0.25 | 0.44 | 1.13 | -0.72 | physical_56 | 0.26 | 0.44 | 1.11 | -0.76 |
| physical_6 | 0.21 | 0.41 | 1.41 | -0.01 | physical_57 | 0.15 | 0.36 | 1.91 | 1.66 |
| physical_7 | 0.24 | 0.43 | 1.19 | -0.56 | physical_58 | 0.23 | 0.42 | 1.29 | -0.31 |
| physical_8 | 0.14 | 0.35 | 2.01 | 2.07 | physical_59 | 0.21 | 0.41 | 1.44 | 0.08 |
| physical_9 | 0.12 | 0.32 | 2.34 | 3.50 | physical_60 | 0.11 | 0.32 | 2.45 | 2.04 |
| physical_10 | 0.20 | 0.40 | 1.51 | 0.29 | physical_61 | 0.30 | 0.46 | 0.88 | -1.21 |
| physical_11 | 0.23 | 0.42 | 1.27 | -0.36 | social_1 | 0.07 | 0.26 | 3.37 | 3.41 |
| physical_12 | 0.11 | 0.32 | 2.43 | 3.91 | social_2 | 0.04 | 0.20 | 2.70 | 2.17 |
| physical_13 | 0.18 | 0.39 | 1.66 | 0.76 | social_3 | 0.09 | 0.29 | 2.84 | 2.11 |
| physical_14 | 0.44 | 0.50 | 0.23 | -1.94 | social_4 | 0.13 | 0.34 | 1.20 | 2.86 |
| physical_15 | 0.47 | 0.50 | 0.11 | -1.98 | social_5 | 0.45 | 0.50 | 0.18 | -1.96 |
| physical_16 | 0.14 | 0.34 | 2.12 | 2.50 | social_6 | 0.35 | 0.48 | 0.61 | -1.62 |
| physical_17 | 0.26 | 0.44 | 1.08 | -0.82 | social_7 | 0.25 | 0.43 | 1.16 | -0.63 |
| physical_18 | 0.37 | 0.48 | 0.54 | -1.70 | social_8 | 0.15 | 0.36 | 1.94 | 1.77 |
| physical_19 | 0.16 | 0.36 | 1.89 | 1.60 | social_9 | 0.16 | 0.37 | 1.81 | 1.28 |
| physical_20 | 0.31 | 0.46 | 0.84 | -1.28 | social_10 | 0.55 | 0.50 | -0.21 | -1.95 |
| physical_21 | 0.13 | 0.34 | 2.22 | 2.93 | social_11 | 0.05 | 0.22 | 3.01 | 3.10 |
| physical_22 | 0.43 | 0.50 | 0.29 | -1.91 | social_12 | 0.18 | 0.39 | 1.63 | .680 |
| physical_23 | 0.59 | 0.49 | -0.35 | -1.87 | social_13 | 0.21 | 0.40 | 1.45 | 0.12 |
| physical_24 | 0.26 | 0.44 | 1.10 | -0.775 | social_14 | 0.33 | 0.47 | 0.70 | -1.51 |
| physical_25 | 0.12 | 0.32 | 2.38 | 3.66 | social_15 | 0.26 | 0.44 | 1.06 | -0.86 |
| physical_26 | 0.10 | 0.30 | 2.65 | 2.03 | social_16 | 0.39 | 0.49 | 0.46 | -1.78 |
| physical_27 | 0.27 | 0.45 | 1.01 | -.967 | social_17 | 0.27 | 0.44 | 1.05 | -0.89 |
| physical_28 | 0.05 | 0.22 | 2.18 | 3.53 | social_18 | 0.19 | 0.39 | 1.58 | 0.51 |
| physical_29 | 0.32 | 0.47 | 0.77 | -1.40 | social_19 | 0.06 | 0.24 | 3.65 | 1.39 |
| physical_30 | 0.18 | 0.39 | 1.62 | 0.64 | social_20 | 0.19 | 0.39 | 1.62 | 0.63 |
| physical_31 | 0.14 | 0.35 | 2.10 | 2.43 | social_21 | 0.24 | 0.43 | 1.21 | -0.52 |
| physical_32 | 0.39 | 0.49 | 0.44 | -1.80 | social_22 | 0.40 | 0.49 | 0.40 | -1.83 |
| physical_33 | 0.10 | 0.30 | 2.69 | 3.28 | social_23 | 0.35 | 0.48 | 0.63 | -1.59 |
| physical_34 | 0.16 | 0.36 | 1.90 | 1.63 | social_24 | 0.08 | 0.28 | 3.00 | 2.05 |
| physical_35 | 0.30 | 0.46 | 0.85 | -1.27 | social_25 | 0.08 | 0.27 | 3.14 | 1.92 |
| physical_36 | 0.27 | 0.45 | 1.02 | -.94 | social_26 | 0.09 | 0.29 | 2.80 | 2.86 |
| physical_37 | 0.55 | 0.50 | -0.19 | -1.94 | social_27 | 0.33 | 0.47 | 0.70 | -1.50 |
| physical_38 | 0.08 | 0.27 | 3.19 | 3.20 | social_28 | 0.12 | 0.32 | 2.38 | 2.68 |
| physical_39 | 0.16 | 0.36 | 1.87 | 1.53 | social_29 | 0.19 | 0.39 | 1.61 | 0.61 |
| physical_40 | 0.56 | 0.50 | -0.26 | -1.93 | social_30 | 0.27 | 0.44 | 1.06 | -0.87 |
| physical_41 | 0.50 | 0.50 | 0.07 | -2.01 | social_31 | 0.39 | 0.49 | 0.45 | -1.79 |
| physical_42 | 0.40 | 0.49 | 0.39 | -1.84 | social_32 | 0.06 | 0.23 | 2.87 | 1.04 |
| physical_43 | 0.17 | 0.37 | 1.79 | 1.21 | social_33 | 0.57 | 0.49 | -0.30 | -1.91 |
| physical_44 | 0.26 | 0.44 | 1.09 | -0.81 | social_34 | 0.13 | 0.33 | 2.24 | 3.05 |
| physical_45 | 0.20 | 0.40 | 1.49 | 0.24 | social_35 | 0.31 | 0.46 | 0.81 | -1.34 |
| physical_46 | 0.40 | 0.49 | 0.41 | -1.83 | social_36 | 0.21 | 0.41 | 1.42 | 0.03 |
| physical_47 | 0.23 | 0.42 | 1.27 | -2.37 | social_37 | 0.34 | 0.47 | 0.67 | -1.55 |
| physical_48 | 0.38 | 0.48 | 0.51 | -1.73 | social_38 | 0.28 | 0.45 | 0.96 | -1.07 |
| physical_49 | 0.10 | 0.31 | 2.58 | 2.66 | social_39 | 0.14 | 0.35 | 2.10 | 2.41 |
| physical_50 | 0.26 | 0.44 | 1.12 | -0.73 | social_40 | 0.17 | 0.38 | 1.74 | 1.05 |
| physical_51 | 0.10 | 0.30 | 2.62 | 2.89 |  |  |  |  |  |

**Table S2. The Descriptive results of the Revised Physical and Social Anhedonia Scale in clinical sample**

| Item | Mean | SD | Skew | Kurt | Item | Mean | SD | Skew | Kurt |
| --- | --- | --- | --- | --- | --- | --- | --- | --- | --- |
| physical_1 | 0.61 | 0.48 | -0.47 | -1.78 | physical_52 | 0.41 | 0.49 | 0.36 | -1.87 |
| physical_2 | 0.46 | 0.49 | 0.17 | -1.98 | physical_53 | 0.33 | 0.47 | 0.73 | -1.47 |
| physical_3 | 0.63 | 0.48 | -.054 | -1.71 | physical_54 | 0.16 | 0.36 | 1.88 | 1.56 |
| physical_4 | 0.51 | 0.50 | -0.02 | -2.01 | physical_55 | 0.39 | 0.48 | 0.43 | -1.89 |
| physical_5 | 0.36 | 0.48 | 0.59 | -1.65 | physical_56 | 0.48 | 0.50 | 0.06 | -2.00 |
| physical_6 | 0.28 | 0.44 | 0.98 | -1.02 | physical_57 | 0.79 | 0.40 | -1.42 | 0.03 |
| physical_7 | 0.78 | 0.41 | -1.34 | -0.20 | physical_58 | 0.70 | 0.45 | -0.88 | -1.23 |
| physical_8 | 0.33 | 0.47 | 0.74 | -.45 | physical_59 | 0.70 | 0.45 | -0.89 | -1.20 |
| physical_9 | 0.19 | 0.39 | 1.60 | 0.59 | physical_60 | 0.76 | 0.42 | -1.23 | -0.47 |
| physical_10 | 0.26 | 0.43 | 1.09 | -0.79 | physical_61 | 0.65 | 0.47 | -0.60 | -1.63 |
| physical_11 | 0.35 | 0.47 | 0.63 | -1.60 | social_1 | 0.22 | 0.41 | 1.33 | -0.212 |
| physical_12 | 0.17 | 0.38 | 1.72 | 0.99 | social_2 | 0.15 | .36 | 1.91 | 1.67 |
| physical_13 | 0.24 | 0.42 | 1.22 | -0.50 | social_3 | 0.29 | 0.45 | 0.91 | -1.16 |
| physical_14 | 0.49 | 0.50 | 0.02 | -2.01 | social_4 | 0.77 | 0.42 | -1.26 | -0.40 |
| physical_15 | 0.71 | 0.45 | -0.90 | -1.19 | social_5 | 0.43 | 0.49 | 0.26 | -1.94 |
| physical_16 | 0.17 | 0.37 | 1.78 | 1.20 | social_6 | 0.49 | 0.50 | 0.05 | -2.00 |
| physical_17 | 0.38 | 0.48 | 0.50 | -1.75 | social_7 | 0.61 | 0.48 | -0.46 | -1.79 |
| physical_18 | 0.61 | 0.48 | -0.46 | -1.79 | social_8 | 0.65 | 0.47 | -0.61 | -1.63 |
| physical_19 | 0.30 | 0.46 | 0.85 | -1.27 | social_9 | 0.83 | 0.37 | -1.75 | 1.07 |
| physical_20 | 0.64 | 0.48 | -0.59 | -1.65 | social_10 | 0.71 | 0.45 | -0.92 | -1.14 |
| physical_21 | 0.20 | 0.39 | 1.53 | 0.35 | social_11 | 0.88 | 0.32 | -1.40 | 1.80 |
| physical_22 | 0.48 | 0.50 | 0.08 | -1.05 | social_12 | 0.79 | 0.40 | -1.45 | 0.12 |
| physical_23 | 0.38 | 0.48 | 0.47 | -1.78 | social_13 | 0.52 | 0.50 | -0.08 | -2.05 |
| physical_24 | 0.70 | 0.46 | -0.85 | -1.27 | social_14 | 0.60 | 0.49 | -0.39 | -1.85 |
| physical_25 | 0.15 | 0.36 | 1.93 | 1.73 | social_15 | 0.53 | 0.50 | -0.12 | -1.99 |
| physical_26 | 0.21 | 0.40 | 1.41 | 0.01 | social_16 | 0.49 | 0.50 | 0.04 | -2.01 |
| physical_27 | 0.41 | 0.49 | 0.38 | -1.86 | social_17 | 0.59 | 0.49 | -0.35 | -1.88 |
| physical_28 | 0.06 | 0.24 | 1.68 | 1.63 | social_18 | 0.61 | 0.48 | -0.45 | -1.80 |
| physical_29 | 0.66 | 0.47 | -0.66 | -1.56 | social_19 | 0.87 | 0.33 | -2.23 | 2.02 |
| physical_30 | 0.77 | 0.42 | -1.27 | -0.38 | social_20 | 0.62 | 0.48 | -0.50 | -1.75 |
| physical_31 | 0.33 | 0.472 | 0.76 | -1.51 | social_21 | 0.50 | 0.50 | 0.06 | -2.01 |
| physical_32 | 0.46 | 0.499 | 0.16 | -1.98 | social_22 | 0.60 | 0.49 | -0.43 | -1.82 |
| physical_33 | 0.87 | 0.341 | -2.16 | 2.68 | social_23 | 0.60 | 0.49 | -0.43 | -1.82 |
| physical_34 | 0.75 | 0.432 | -1.17 | -0.61 | social_24 | 0.81 | 0.39 | -1.57 | 0.47 |
| physical_35 | 0.63 | 0.484 | -0.53 | -1.72 | social_25 | 0.90 | 0.30 | -2.65 | 2.06 |
| physical_36 | 0.67 | 0.470 | -0.74 | -1.45 | social_26 | 0.28 | 0.45 | 0.98 | -1.03 |
| physical_37 | 0.41 | 0.492 | 0.37 | -1.87 | social_27 | 0.66 | 0.47 | -0.69 | -1.52 |
| physical_38 | 0.86 | 0.351 | -2.04 | 2.17 | social_28 | 0.40 | 0.49 | 0.39 | -1.85 |
| physical_39 | 0.82 | 0.386 | -1.66 | 0.76 | social_29 | 0.38 | 0.48 | 0.49 | -1.76 |
| physical_40 | 0.60 | 0.490 | -0.41 | -1.83 | social_30 | 0.55 | 0.49 | -0.21 | -1.96 |
| physical_41 | 0.48 | 0.500 | 0.08 | -2.05 | social_31 | 0.57 | 0.49 | -0.27 | -1.98 |
| physical_42 | 0.48 | 0.500 | 0.08 | -2.04 | social_32 | 0.22 | 0.41 | 1.35 | -.16 |
| physical_43 | 0.24 | 0.429 | 1.21 | -0.53 | social_33 | 0.49 | 0.50 | 0.04 | -2.01 |
| physical_44 | 0.67 | 0.472 | -0.71 | -1.49 | social_34 | 0.35 | 0.47 | 0.61 | -1.63 |
| physical_45 | 0.71 | 0.455 | -0.92 | -1.15 | social_35 | 0.51 | 0.51 | -0.04 | -2.01 |
| physical_46 | 0.51 | 0.501 | -0.02 | -2.01 | social_36 | 0.58 | 0.49 | -0.31 | -1.90 |
| physical_47 | 0.27 | 0.444 | 1.05 | -0.90 | social_37 | 0.49 | 0.51 | 0.04 | -2.01 |
| physical_48 | 0.64 | 0.482 | -0.56 | -1.69 | social_38 | 0.62 | 0.48 | -0.49 | -1.76 |
| physical_49 | 0.13 | 0.338 | 2.19 | 2.83 | social_39 | 0.37 | 0.48 | 0.53 | -1.72 |
| physical_50 | 0.31 | 0.465 | 0.80 | -1.36 | social_40 | 0.42 | 0.49 | 0.31 | -1.91 |
| physical_51 | 0.18 | 0.383 | 1.69 | 0.86 |  |  |  |  |  |

**Table S3. Factor Loadings for the Item-Level in EFAs of the RPAS and RSAS**

| Item | Factor1 (physical consummatory anhedonia) | Factor2 (physical anticipatory anhedonia) | Item | Factor1  (social  consummatory anhedonia) | Factor2  (social anticipatory anhedonia) |
| --- | --- | --- | --- | --- | --- |
| physical_5 | 0.497 |  | social_1 | 0.66 |  |
| physical_6 | 0.477 |  | social_2 | 0.698 |  |
| physical_8 | 0.600 |  | social_3 | 0.650 |  |
| physical_10 | 0.510 |  | social_6 | 0.289 |  |
| physical_11 | 0.421 |  | social_10 | 0.433 |  |
| physical_12 | 0.463 |  | social_13 | 0.510 |  |
| physical_13 | 0.526 |  | social_14 | 0.499 |  |
| physical_14 | 0.525 |  | social_17 | 0.644 |  |
| physical_15 | 0.448 |  | social_21 | 0.581 |  |
| physical_16 | 0.450 |  | social_22 | 0.566 |  |
| physical_17 | 0.646 |  | social_23 | 0.636 |  |
| physical_18 | 0.604 |  | social_26 | 0.656 |  |
| physical_20 | 0.539 |  | social_27 | 0.623 |  |
| physical_22 | 0.640 |  | social_28 | 0.713 |  |
| physical_23 | 0.435 |  | social_29 | 0.503 |  |
| physical_26 | 0.654 |  | social_32 | 0.655 |  |
| physical_27 | 0.689 |  | social_34 | 0.703 |  |
| physical_28 | 0.403 |  | social_35 | 0.441 |  |
| physical_29 | 0.758 |  | social_37 | 0.409 |  |
| physical_32 | 0.541 |  | social_38 | 0.685 |  |
| physical_33 | 0.289 |  | social_39 | 0.725 |  |
| physical_43 | 0.308 |  | social_40 | 0.629 |  |
| physical_44 | 0.558 |  | social_4 |  | 0.671 |
| physical_48 | 0.604 |  | social_5 |  | 0.463 |
| physical_50 | 0.669 |  | social_7 |  | 0.596 |
| physical_51 | 0.474 |  | social_8 |  | 0.680 |
| physical_52 | 0.586 |  | social_9 |  | 0.612 |
| physical_55 | 0.751 |  | social_11 |  | 0.813 |
| physical_56 | 0.547 |  | social_12 |  | 0.494 |
| physical_57 | 0.527 |  | social_15 |  | 0.680 |
| physical_2 |  | 0.305 | social_16 |  | 0.505 |
| physical_3 |  | 0.491 | social_18 |  | 0.614 |
| physical_7 |  | 0.389 | social_19 |  | 0.814 |
| physical_19 |  | 0.461 | social_20 |  | 0.601 |
| physical_21 |  | 0.494 | social_24 |  | 0.771 |
| physical_24 |  | 0.370 | social_25 |  | 0.790 |
| physical_25 |  | 0.544 | social_30 |  | 0.549 |
| physical_30 |  | 0.501 | social_31 |  | 0.470 |
| physical_31 |  | 0.628 | social_36 |  | 0.642 |
| physical_34 |  | 0.756 |  |  |  |
| physical_35 |  | 0.725 |  |  |  |
| physical_36 |  | 0.486 |  |  |  |
| physical_37 |  | 0.456 |  |  |  |
| physical_38 |  | 0.471 |  |  |  |
| physical_39 |  | 0.721 |  |  |  |
| physical_41 |  | 0.649 |  |  |  |
| physical_42 |  | 0.363 |  |  |  |
| physical_45 |  | 0.576 |  |  |  |
| physical_46 |  | 0.648 |  |  |  |
| physical_47 |  | 0.472 |  |  |  |
| physical_49 |  | 0.324 |  |  |  |
| physical_54 |  | 0.503 |  |  |  |
| physical_58 |  | 0.583 |  |  |  |
| physical_59 |  | 0.681 |  |  |  |
| physical_60 |  | 0.765 |  |  |  |
| physical_61 |  | 0.596 |  |  |  |

**Table S4. Factor Loading of the Revised Physical Anhedonia Scale in EFA**

| Item | Factor1 | Factor2 | Factor3 | Factor4 | Item | Factor1 | Factor2 | Factor3 | Factor4 |
| --- | --- | --- | --- | --- | --- | --- | --- | --- | --- |
| physical_2 |  | 0.305 |  |  | physical_32 | 0.541 |  |  |  |
| physical_3 |  | 0.491 |  |  | physical_33 | 0.289 |  |  |  |
| physical_5 | 0.497 |  |  |  | physical_34 |  | 0.756 |  |  |
| physical_6 | 0.477 |  |  |  | physical_35 |  | 0.725 |  |  |
| **physical_7** |  | 0.389 | 0.510 |  | physical_36 |  | 0.486 |  |  |
| physical_8 | 0.600 |  |  |  | physical_37 |  | 0.456 |  |  |
| physical_10 | 0.510 |  |  |  | **physical_38** |  | 0.471 |  | 0.562 |
| physical_11 | 0.421 |  |  |  | physical_39 |  | 0.721 |  |  |
| physical_12 | 0.463 |  |  |  | physical_41 |  | 0.649 |  |  |
| physical_13 | 0.526 |  |  |  | **physical_42** |  | 0.363 |  | 0.376 |
| physical_14 | 0.525 |  |  |  | physical_43 | 0.308 |  |  |  |
| **physical_15** | 0.448 |  | 0.521 |  | physical_44 | 0.558 |  |  |  |
| physical_16 | 0.450 |  |  |  | physical_45 |  | 0.576 |  |  |
| physical_17 | 0.646 |  |  |  | physical_46 |  | 0.648 |  |  |
| physical_18 | 0.604 |  |  |  | physical_47 |  | 0.472 |  |  |
| physical_19 |  | 0.461 |  |  | physical_48 | 0.604 |  |  |  |
| physical_20 | 0.539 |  |  |  | physical_49 |  | 0.324 |  |  |
| physical_21 |  | 0.494 |  |  | physical_50 | 0.669 |  |  |  |
| physical_22 | 0.640 |  |  |  | physical_51 | 0.474 |  |  |  |
| physical_23 | 0.435 |  |  |  | physical_52 | 0.586 |  |  |  |
| physical_24 |  | 0.370 |  |  | physical_54 |  | 0.503 |  |  |
| physical_25 |  | 0.544 |  |  | physical_55 | 0.751 |  |  |  |
| physical_26 | 0.654 |  |  |  | physical_56 | 0.547 |  |  |  |
| physical_27 | 0.689 |  |  |  | physical_57 | 0.527 |  |  |  |
| physical_28 | 0.403 |  |  |  | physical_58 |  | 0.583 |  |  |
| physical_29 | 0.758 |  |  |  | physical_59 |  | 0.681 |  |  |
| physical_30 |  | 0.501 |  |  | physical_60 |  | 0.765 |  |  |
| physical_31 |  | 0.628 |  |  | physical_61 |  | 0.596 |  |  |
